# CEBPG transcription factor correlates with antioxidant and DNA repair genes in normal bronchial epithelial cells but not in individuals with bronchogenic carcinoma

**DOI:** 10.1186/1471-2407-5-141

**Published:** 2005-10-29

**Authors:** D'Anna N Mullins, Erin L Crawford, Sadik A Khuder, Dawn-Alita Hernandez, Youngsook Yoon, James C Willey

**Affiliations:** 1 Department of Medicine, Medical University of Ohio, Room 0012 Ruppert Health Building, 3000 Arlington Avenue, Toledo, OH 43614, USA

## Abstract

**Background:**

Cigarette smoking is the primary cause of bronchogenic carcinoma (BC), yet only 10–15% of heavy smokers develop BC and it is likely that this variation in risk is, in part, genetically determined. We previously reported a set of antioxidant genes for which transcript abundance was lower in normal bronchial epithelial cells (NBEC) of BC individuals compared to non-BC individuals. In unpublished studies of the same NBEC samples, transcript abundance values for several DNA repair genes were correlated with these antioxidant genes. From these data, we hypothesized that antioxidant and DNA repair genes are co-regulated by one or more transcription factors and that inter-individual variation in expression and/or function of one or more of these transcription factors is responsible for inter-individual variation in risk for BC.

**Methods:**

The putative transcription factor recognition sites common to six of the antioxidant genes were identified through in silico DNA sequence analysis. The transcript abundance values of these transcription factors (n = 6) and an expanded group of antioxidant and DNA repair genes (n = 16) were measured simultaneously by quantitative PCR in NBEC of 24 non-BC and 25 BC individuals.

**Results:**

CEBPG transcription factor was significantly (p < 0.01) correlated with eight of the antioxidant or DNA repair genes in non-BC individuals but not in BC individuals. In BC individuals the correlation with CEBPG was significantly (p < 0.01) lower than that of non-BC individuals for four of the genes (XRCC1, ERCC5, GSTP1, and SOD1) and the difference was nearly significant for GPX1. The only other transcription factor correlated with any of these five target genes in non-BC individuals was E2F1. E2F1 was correlated with GSTP1 among non-BC individuals, but in contrast to CEBPG, there was no significant difference in this correlation in non-BC individuals compared to BC individuals.

**Conclusion:**

We conclude that CEBPG is the transcription factor primarily responsible for regulating transcription of key antioxidant and DNA repair genes in non-BC individuals. Further, we conclude that the heavy smokers selected for development of BC are those who have sub-optimal regulation of antioxidant and DNA repair genes by CEBPG.

## Background

BC is currently the leading cause of cancer-related death in the United States, causing 28% of all cancer deaths [1]. Although cigarette smoking is the primary risk factor, only 10–15% of heavy smokers (greater than 20 pack years) develop BC [1-3]. Antioxidant and DNA repair enzymes that provide protection from the effects of cigarette smoke are expressed in the progenitor cells for BC, normal bronchial epithelial cells (NBEC) [1]. Inherited inter-individual variation in the function of these genes plays a role in determining risk for BC [4-6]. Antioxidant enzymes protect NBEC from reactive oxygen species produced by interaction with and metabolism of xenobiotics such as pollution and cigarette smoke [4-7] as well as those produced by normal cellular metabolism. Reactive oxygen species cause many damaging reactions including denaturation of proteins, cross-linking of lipids and proteins and modification of nucleic acid bases, which can lead to cancer [7]. DNA repair enzymes repair the frequent damage to DNA caused by oxidant stress as well as other stresses, including bulky adducts derived from carcinogens in cigarette smoke [8].

We previously reported that an interactive transcript abundance index comprising antioxidant genes was lower in NBEC of BC individuals compared to non-BC individuals, suggesting that BC individuals are selected on the basis of poor antioxidant protection [9]. In that study, there was a tendency towards correlation in transcript abundance between several pairs of antioxidant or DNA repair genes in non-BC individuals, but not in BC individuals. Gene pairs included in that observation were GSTP1/GPX1, CAT/GPX3, and GPX3/SOD1.

Correlation is one typical characteristic of co-regulated genes. Another is shared transcription factor recognition sites in the regulatory regions of those genes [10]. Based on the above findings, it was hypothesized first, that there is inter-individual variation in regulation of key antioxidant and DNA repair genes by one or more transcription factors and second, that individuals with sub-optimal regulation are selected for development of BC if they smoke cigarettes. To test these hypotheses, transcription factor recognition sites common to the regulatory regions of the above correlated gene pairs were identified through in silico DNA sequence analysis, and their transcript abundance measured simultaneously with an expanded group of ten antioxidant and six DNA repair genes.

## Methods

### NBEC sample procurement

Brush biopsy samples of normal bronchial epithelium were obtained for research studies at the time of diagnostic bronchoscopy according to previously described methods [9,11]. Normal bronchial epithelium in the lung not involved with cancer was brushed prior to biopsy of the suspected cancerous area. Samples were collected in a manner satisfying all requirements of the Institutional Review Board for the Medical University of Ohio. Each BC diagnosis and subtype identification was determined by histopathological examination in the Department of Pathology at the Medical University of Ohio. NBEC samples from a total of 49 individuals, including 24 non-BC individuals and 25 BC individuals, were evaluated in this study. The biographical characteristics of these individuals are presented in Table 1.

### Transcript abundance measurement

Total RNA samples extracted from NBEC were reverse transcribed using M-MLV reverse transcriptase and oligo dT primers as previously described [9,11]. Standardized RT (StaRT)-PCR was used for transcript abundance measurement in these studies. With StaRT-PCR, an internal standard for each gene within a standardized mixture of internal standards (SMIS) is included in each PCR reaction. After amplification, products were electrophoresed on an Agilent 2100 Bioanalyzer using DNA Chips with DNA 1000 Kit reagents for visualization according to the manufacturer's protocol (Agilent Technologies Deutschland GmbH, Waldbronn, Germany).

The StaRT-PCR technology is licensed to Gene Express, Inc. (Toledo, OH). Many of the reagents are available commercially and were obtained through Gene Express, Inc. for this study. StaRT-PCR reagents for each of the measured genes that were not commercially available, including primers and SMIS, were prepared according to previously described methods [11,12]. Sequence information for the primers is provided in Table 2.

Including an internal standard within a SMIS in each measurement controls for all known sources of variation during PCR, including inhibitors in samples, and generates virtually-multiplexed transcript abundance data that are directly comparable across multiple experiments and institutions [13]. The performance characteristics of StaRT-PCR are superior to other forms of commercially available quantitative PCR technology in the areas critical to this study. With respect to these studies, the key property of a quantitative PCR method is not whether the PCR products are measured kinetically or at endpoint, but rather whether there are internal standards in each measurement or not. The overall performance characteristics of StaRT-PCR, including extensive validation of the method in independent laboratories have been presented in several recent articles and chapters [13-15]. With respect to the genes measured in this study, for each gene the StaRT-PCR reagents had lower detection threshold of less than 10 molecules, linear dynamic range of more than six orders of magnitude (less than 10 to over 10^7 ^molecules), and signal-to-analyte response of 100%. In addition, the presence of an internal standard controls for inter-sample variation in presence of PCR inhibitors (which often are gene-specific) and ensures no false negatives (if the PCR fails the internal standard PCR product is not observed and there are no data to report). False positives are eliminated through use of a control PCR reaction with no cDNA in it.

### Statistical analysis

More than 6,000 transcript abundance measurements were conducted in multiple experiments over two years to assess the six transcription factors and sixteen antioxidant and DNA repair genes in NBEC samples from 49 individuals (24 non-BC individuals and 25 BC individuals).

Correlation of each of the six transcription factors with each of the antioxidant or DNA repair genes was determined by Pearson's correlation following logarithmic transformation. The transformation was necessary due to the wide biological variation in expression of each gene among the individuals. Significance level was defined as p < 0.01 following Bonferroni adjustment for multiple comparison, specifically comparison of each of six transcription factors to each of the antioxidant or DNA repair genes. Comparison for significant differences between pairs of correlation coefficients was done by Fisher's Z-transformation test [16].

Analysis of the relationship between virtually-multiplexed transcript abundance data for each gene with age was assessed by Pearson's correlation, with gender by t-test, and with smoking history by ANOVA followed by Duncan's test.

### Transcription factor recognition site analysis

The El Dorado (Build 35) program from the Genomatix software package was used to locate the correlated genes within the genome and define 1101 base pairs of the promoter regions (1000 base pairs upstream of and 100 base pairs into the transcription start site) for each gene (Genomatix Software GmbH, Munich, Germany, [17]). The 1101 base pair sequences obtained from the El Dorado program then were used as the target sequences for putative transcription factor recognition site identification using the MatInspector Version 4.2 program, which yielded sites for 11 transcription factors (Genomatix Software GmbH, Munich, Germany, [17]). The parameters used were the standard (0.75) core similarity and the optimized matrix similarity [18]. StaRT-PCR reagents were optimized for ten of these transcription factors, including CEBPB, CEBPE, CEBPG, E2F1, E2F3, E2F4, E2F5, E2F6, EVI1, and PAX5. Four transcription factors were expressed at low and invariant levels among multiple NBEC samples and were therefore excluded from the study. The remaining six, CEBPB, CEBPG, E2F1, E2F3, E2F6, and EVI, were evaluated for correlation with an expanded group of ten antioxidant and six DNA repair genes.

## Results

Virtually-multiplexed transcript abundance data were obtained for each gene in each of the 49 samples, except for E2F1 measurement in sample 147 (Table 3). A gene-specific inhibitor in sample 147 prevented amplification of E2F1. Neither the internal standard, nor the native cDNA PCR product was observed. The presence of gene-specific PCR inhibition was observable in some other samples as reduction in peak heights in internal standard PCR products relative to that expected for the number of internal standard molecules present at the beginning of the PCR reaction. However, in each such case, the PCR amplification was efficient enough to enable quantification.

### Bivariate analysis

In non-BC individuals there was significant (p < 0.01) correlation between CEBPG and eight of the 16 antioxidant or DNA repair genes, specifically XRCC1, ERCC5, GSTP1, SOD1, GPX1, ERCC1, CAT and ERCC2 (Table 4). In contrast, in BC individuals samples CEBPG was not correlated with any of the antioxidant or DNA repair genes. These relationships were not observed with any of the other transcription factors studied.

**Figure 1:**
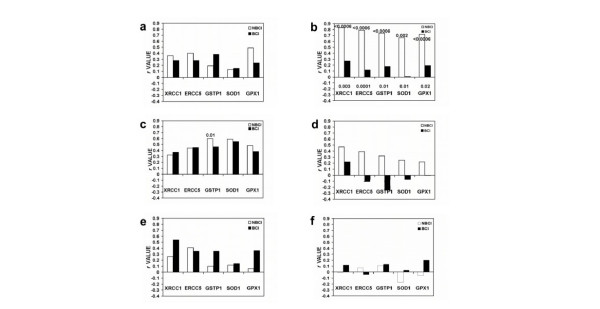
**Correlation of each transcription factor with XRCC1, ERCC5, GSTP1, SOD1, or GPX1**. **(a-f) **Each panel presents the correlation coefficients (*r *values) for one transcription factor in relation to each of the five genes: **(a) **CEBPB, **(b) **CEBPG, **(c) **E2F1, **(d) **E2F3, **(e) **E2F6, **(f) **EVI1. The p value for each significant correlation is provided above the bar. For CEBPG, presented in panel **b**, the difference in *r *value between non-BC individuals and BC individuals was significant or nearly significant for each correlated gene, and the p value for each comparison is provided below the corresponding pair of bars.

**Figure 2:**
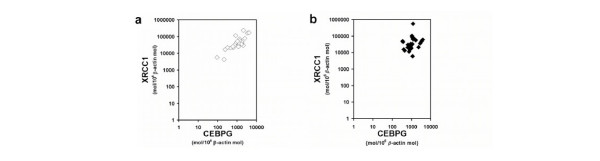
**Scatter plot representation of bivariate correlation of CEBPG with XRCC1**. **(a, b) **CEBPG/XRCC1 data from Figure 1**b **presented as scatter plots: **(a) **non-BC individuals, **(b) **BC individuals.

For XRCC1, ERCC5, GSTP1, and SOD1 the correlation with CEBPG was significantly lower in BC individuals compared to non-BC individuals and the difference was nearly significant for GPX1 (Fig. 1b). Scatter plots of the relationship between CEBPG and XRCC1 in non-BC individuals or BC individuals (Fig. 2a,b) are representative of the other four genes. Neither CEBPG, nor XRCC1, ERCC5, GSTP1, SOD1 or GPX1 was significantly correlated with age, gender, or smoking history in non-BC individuals, BC individuals, or the combined group.

In non-BC individuals, based on the *r*^2 ^values from Pearson's correlation analysis, CEBPG accounts for much of the variance in expression of XRCC1 (69%), ERCC5 (62%), GSTP1 (55%), SOD1 (44%), and GPX1 (52%). E2F1 accounts for some of the remaining variance. For example, when samples from all 49 non-BC individuals and BC individuals were assessed as a single group, E2F1 was significantly correlated with ERCC5, GSTP1 and SOD1 (Table 4). Further, in non-BC individuals, E2F1 was correlated with GSTP1 (Fig. 1c) and the correlation was lower in BC individuals. However, the difference in correlation between non-BC individuals and BC individuals was not significant. None of the other transcription factors were correlated with XRCC1, ERCC5, GSTP1, SOD1, or GPX1 (Fig. 1a,d,e,f).

### Comparison of gene expression with demographic characteristics

E2F1 and GSTZ1 each were positively correlated with age. GSTM1-5 was the only gene with a difference in expression by gender. There was a difference in ERCC2 expression between former and never smokers.

## Discussion

In this study, we tested two hypotheses. First, that there is inter-individual variation in regulation of key antioxidant and DNA repair genes by one or more transcription factors. Second, that individuals with sub-optimal regulation are selected for development of BC if they smoke cigarettes.

These hypotheses are supported by the findings that a) there was large inter-individual variation in transcript levels of CEBPG and each of the target genes and in non-BC individuals, b) CEBPG transcript abundance values were significantly correlated by bivariate analysis with the transcript abundance values of four key antioxidant and DNA repair genes in non-BC individuals, and c) that there was no correlation between CEBPG and these genes in BC individuals.

These results support the hypothesis that each of the antioxidant or DNA repair genes correlated with CEBPG in non-BC individuals is regulated by CEBPG. This is supported by the specificity of the CEBPG correlation. That is, there was lack of correlation between any of the other five transcription factors assessed and these target genes. Of particular note is the lack of correlation of the target genes with CEBPB, which binds to the same recognition site as CEBPG, and shares its recognition site within each of the antioxidant or DNA repair genes. However, there are alternative explanations for the observed correlation of CEBPG with antioxidant and DNA repair genes in non-BC individuals. One possibility is that CEBPG and each of the correlated antioxidant or DNA repair genes is regulated by a transcription factor that is as yet undiscovered, and/or has a recognition site that is not yet known and was not in the Genomatix software database.

There also is more than one possible explanation for the observed lack of correlation between CEBPG and antioxidant or DNA repair genes in BC individuals. For example, the non-BC individual and BC individual groups are not perfectly matched with respect to age, gender or smoking history (Table 1) and each of these factors could contribute to the observed difference in correlation between groups. However, the lack of association of transcript abundance level for CEBPG, XRCC1, ERCC5, GSTP1, SOD1, or GPX1 with age, gender or smoking history argues against such an explanation. One way to examine this possibility is through additional, larger, more closely matched studies. Another possible explanation is that any differences in NBEC from BC individuals compared to non-BC individuals resulted from development of BC, instead of being a hereditary cause of increased risk for cancer. The best way to determine this will be to conduct a prospective study. In such a study, individuals matched for smoking history will be monitored for development of BC over time. The correlation of transcript abundance values for CEBPG relative to transcript abundance values for each of the antioxidant or DNA repair genes will be assessed. It is expected that the greatest incidence of BC will be among the heaviest smokers. Among the matched heaviest smokers, it is expected that CEBPG will be significantly correlated with each of the antioxidant or DNA repair genes among the non-BC individuals but not correlated in BC individuals.

Thus, there are multiple possible explanations for the observed findings. However, based on the preponderance of data thus far available, we conclude that CEBPG is responsible for optimal transcriptional regulation of key antioxidant or DNA repair genes in NBEC and that there is inter-individual variation in the regulation of each of these genes by CEBPG. If this conclusion is correct, the individuals at greatest risk for BC will be those with the most extreme smoking history combined with sub-optimal regulation of the largest number of antioxidant and DNA repair genes. This, in turn, leads to increased representation among BC individuals of individuals with lack of correlation between CEBPG and each of the affected antioxidant and/or DNA repair genes.

CEBPG is a truncated CEBP transcription factor [19] and possesses the sequences necessary for DNA binding and heterodimer formation, but lacks the sequences necessary for transactivation [20]. CEBPG forms heterodimers with other CEBP family members and in other tissues this leads to increased [21] or decreased [20] transcription of the regulated gene. CEBPG is known to have stimulatory effect on the IL-6 and IL-8 promoters in B cell lines [21], and can also act as a dominant negative regulator of CEBPA and CEBPB in fibroblast and B cell lines [20].

The data from CEBPG knockout mice support a role for CEBPG in protecting lungs from oxidant damage. CEBPG^-/- ^knockout mice are healthy at birth but begin to die within 24 hours, and histological examination reveals emphysematous lungs [22]. In humans, risk for emphysema is associated with antioxidant capacity [23], and there is a strong correlation between risk for emphysema and risk for BC.

However, it will be important to obtain direct experimental evidence in NBECs for the role of CEBPG in regulating the antioxidant and DNA repair genes included in this study. Correlation between CEBPG and target gene transcript levels may not be associated with correlation at the protein level.

In this study, E2F1 correlation with DNA repair and antioxidant genes was less than the correlation observed with CEBPG, and the E2F1 correlation was observed in both non-BC individuals as well as BC individuals. The maintained correlation of E2F1 with DNA repair and antioxidant genes in BC individuals suggests that this function is more tightly controlled in the population and does not play a role in determination of risk for BC. E2F1 has previously been reported to regulate transcription of DNA repair enzyme genes in other cell types, including primary human fibroblasts and mouse epidermal cells [24,25]. Clearly this would have survival value since DNA repair gene up-regulation in response to E2F1 provides additional DNA repair when the DNA is replicating and is particularly vulnerable to damage.

Epidemiologic assessment of the correlation between a particular variation in DNA sequence, or polymorphism, and risk for BC has been a dominant paradigm for many years. Thus far, these efforts have met with scant success [26]. A common limitation in design of such studies is that they involve assessment of a single polymorphism or occasionally, a few polymorphisms. Further, although the polymorphism assessed typically resides within a gene known to protect bronchial epithelium from carcinogens, oxidants, or DNA damage, the selection of the particular polymorphism for study is largely empiric, and not based on known functional properties. These are problems because multiple infrequent polymorphisms at different sites may all contribute to risk and unless the key polymorphisms can be identified through a functional test, a statistically valid assessment would require much larger study populations [27].

The findings of this study support a novel approach to identifying clinically useful biomarkers. According to the paradigm used in this study, a) a normal phenotype results from regulated transcription of a group of genes by one or more transcription factors, b) the corresponding risk-conferring or disease phenotype results from sub-optimal interaction among those same genes, and c) each phenotype is identifiable and distinguishable through virtually-multiplexed transcript abundance analysis. The data presented here support the utility of this paradigm in identifying genes associated with risk for BC.

The next step will be to identify polymorphisms that affect regulation of XRCC1, ERCC5, GSTP1, SOD1, and GPX1 by CEBPG. Such polymorphisms should yield biomarkers suitable for more readily accessible samples, such as peripheral blood or buccal smears. A biomarker combining polymorphisms that affect regulation with those that affect function of antioxidant and DNA repair genes is likely to be the most accurate for identifying individuals at risk for BC. Biomarkers that accurately identify individuals at risk for BC will improve efficacy of chemoprevention and early detection clinical trials.

The observed inter-sample variation in the presence of gene-specific inhibitors of PCR provides evidence supporting the need for inclusion of an internal standard in each quantitative PCR transcript abundance measurement. Including such internal standards in the form of standardized mixtures of internal standards improves the reproducibility of transcript abundance measurement and enables development of a standardized database comprising virtually-multiplexed transcript abundance data. Virtually-multiplexed transcript abundance data are highly suited to identification of genes that have correlated transcript abundance values. Correlation at the transcript abundance level is an important property of genes that are co-regulated at the transcription level.

## Conclusion

We conclude that in non-BC individuals, CEBPG regulates transcription of key antioxidant or DNA repair genes in NBEC and that in smokers who develop BC, CEBPG regulation is sub-optimal for a sufficient number of antioxidant and/or DNA repair genes to cause increased risk.

## Competing interests

JCW and ELC each have significant equity interest in Gene Express, Inc., which produces and markets StaRT-PCR™ reagents used in this study.

## Authors' contributions

DNM participated in the design of the study, performed the TF identification, carried out TA measurements, coordinated and participated in the statistical analysis and drafted the manuscript. ELC contributed the preliminary data, participated in the design of the study and carried out TA measurements. SAK performed the statistical analysis for interpretation of data. DAH and YY consented patients and obtained the primary samples necessary for the study according to IRB regulations. JCW conceived of the study, participated in its design and coordination, and helped to draft the manuscript. All authors read and approved the final manuscript.

## Pre-publication history

The pre-publication history for this paper can be accessed here:



## Figures and Tables

**Table 1:** Demographic data of patients from whom the NBEC samples were obtained.

**Subject #**	**Group**	**Age**	**Gender**	**Histology**	**Smoking Hx**^1^	**Ethnicity**
63	NBCI^2^	77	M		75	W^3^
64	NBCI	47	F		45	W
136	NBCI	38	M		25	A.A.^4^
139	NBCI	44	F		17.5	W
150	NBCI	70	F		45	W
156	NBCI	46	F		NS^5^	A.A.
157	NBCI	60	M		>100	W
194	NBCI	57	M		3	W
210	NBCI	40	M		34	W
257	NBCI	69	F		20	W
261	NBCI	73	F		NS	W
282	NBCI	83	F		60	W
285	NBCI	69	F		NS	W
296	NBCI	43	F		20	W
305	NBCI	50	F		40	W
315	NBCI	64	M		N/A^6^	W
330	NBCI	39	M		NS	W
331	NBCI	N/A	N/A		N/A	N/A
334	NBCI	51	M		>50	W
336	NBCI	31	F		NS	W
337	NBCI	32	M		22	N/A
339	NBCI	59	M		50	W
361	NBCI	73	F		NS	H^7^
363	NBCI	50	M		20	A.A.

34	BCI^8^	80	M	NSCLC^9^	40	W
71	BCI	63	M	NSCLC	100	W
85	BCI	73	F	SQ^10^	>100	W
88	BCI	85	M	SQ	75	W
99	BCI	63	M	NSCLC	45	W
118	BCI	72	M	SQ	30	W
146	BCI	64	F	SCLC^11^	45	W
147	BCI	76	M	SCLC	75	W
158	BCI	88	M	SCLC	115.5	W
167	BCI	60	F	NSCLC	50	W
171	BCI	67	M	SCLC	100	W
191	BCI	75	M	SQ	54	W
211	BCI	71	M	SQ	50	W
212	BCI	65	M	SQ	67.5	W
247	BCI	75	F	SQ	50	W
255	BCI	60	F	NSCLC	30	W
259	BCI	68	M	CS^12^	137.5	W
271	BCI	58	M	AC^13^	94.5	W
287	BCI	65	F	NSCLC	50	W
300	BCI	56	M	SQ	34	W
306	BCI	46	M	SQ	30	W
314	BCI	69	F	BC^14^	NS	W
329	BCI	76	F	PD^15^	>37.5	W
335	BCI	75	M	SCLC	58	A.A.
B3	BCI	63	M	SQ	60	W

^1^Pack years; ^2^Non-bronchogenic carcinoma individual; ^3^White; ^4^African-American; ^5^Non-smoker; ^6^Not available; ^7^Hispanic; ^8^Bronchogenic carcinoma individual; ^9^Non-small cell lung cancer; ^10^Squamous carcinoma; ^11^Small cell lung cancer; ^12^Carcinoma-in-situ; ^13^Adenocarcinoma;^14^Bronchogenic Cancer, histology not specified; ^15^Poorly differentiated carcinoma.

**Table 2:** Sequence for each primer used for StaRT-PCR virtually-multiplexed transcript abundance measurement or for internal standard preparation (CT) [15].

**Gene**	**Accession #**	**Primer**	**Sequence**	**Position**	**Product**
ACTB	X00351	Forward	5' ATC CTC ACC CTG AAG TAC CC 3'	231	
		Reverse	5' CCA TCT CTT GCT CGA AGT CC 3'	704	493 bp
		CT	5' CCA TCT CTT GCT CGA AGT CCG CCA GCC AGG TCC AGA CGC A 3'	568	377 bp
CAT	X04076	Forward	5' CCA GAA GAA AGC GGT CAA GA 3'	1492	
		Reverse	5' AAC CTT CAT TTT CCC CTG GG 3'	1822	350 bp
		CT	5' AAC CTT CAT TTT CCC CTG GGC CAG TGA TGA GCG GGT TAC A 3'	1699	247 bp
CEBPB	NM_005194	Forward	5' TGT CCA AAC CAA CCG CAC AT 3'	1412	
		Reverse	5' AGC AAC AAG CCC GTA GGA AC 3'	1657	265 bp
		CT	5' AGC AAC AAG CCC GTA GGA ACA CGC GTT CAG CCA TGT TTA A 3'	1571	199 bp
CEBPG	U20240	Forward	5' CGG TTG AAA AGC AAG CAG AAA GCA 3'	488	
		Reverse	5' GAT CCC AGA AAA TAG CCT CCA ATG 3'	814	350 bp
		CT	5' GAT CCC AGA AAA TAG CCT CCA ATG AAC ATT CAA GCC ACA AGC TC 3'	726	282 bp
E2F1	M96577	Forward	5' TGA TAC CCC AAC TCC CTC TA 3'	2076	
		Reverse	5' AAA GCA GGA GGG AAC AGA GC 3'	2452	396 bp
		CT	5' AAA GCA GGA GGG AAC AGA GCA CTG CAG GGA CCA CAG G 3'	2363	327 bp
E2F3	Y10479	Forward	5' TGA AAG CCC CTC CAG AAA CAA G 3'	1019	
		Reverse	5' GCA GCA GGG GAG GCA GTA AGT T 3'	1336	339 bp
		CT	5' GCA GCA GGG GAG GCA GTA AGT TGG GGA GGC CAG AGG AGA AAG GT 3'	1253	278 bp
E2F6	AF059292	Forward	5' GGG CCT GCT GCC ATC AAA AAT A 3'	99	
		Reverse	5' CCG CTT TCG GAC TCC CAG TTT 3'	283	205 bp
		CT	5' CCG CTT TCG GAC TCC CAG TTA GCG ATA CAT CAA AAC GAG G 3'	184	125 bp
ERCC1	M13194	Forward	5' CTG GAG CCC CGA GGA AGC 3'	739	
		Reverse	5' CAC TGG GGG TTT CCT TTG 3'	1049	328 bp
		CT	5' CAC TGG GGG TTT CCT TGG AAG GCC AGA TCT TCT CTT 3	928	240 bp
ERCC2	X52221	Forward	5' GGC CTT CTT CAC CAG CTA C 3'	1608	
		Reverse	5' GTA GTC CGT CTT GCC CCT G 3'	2004	415 bp
		CT	5' GTA GTC CGT CTT GCC CCT GTG GAA CTG GTC CCG CAG GT 3'	2597	346 bp
ERCC4	U64315	Forward	5' AGT GCA TCT CCA TGT CCC GCT ACT A 3'	2213	
		Reverse	5' CGA TGT TCT TAA CGT GGT GCA TCA A 3'	2578	390 bp
		CT	5' CGA TGT TCT TAA CGT GGT GCA TCA ACA GGC TGT GGC TTG CTT TGT 3'	2433	265 bp
ERCC5	D16305	Forward	5' AAG GAA AGA GAA AGA AGC AGC AGC CA 3'	3087	
		Reverse	5' CAA ACA CAG ATC TGG CGG TCA CGA GG 3'	3501	440 bp
		CT	5' CAA ACA CAG ATC TGG CGG TCA CGA GGA GCT TCC TTC ACT GAG TTC TGC GAA T 3'	3401	366 bp
EVI1	NM_005241	Forward	5' CGC CGG ATA TCC ACG AAG A 3'	302	
		Reverse	5' ATG CTG AGA GCG AAT GTG C 3'	711	428 bp
		CT	5' ATG CTG AGA GCG AAT GTG CTT AAA TGC CTT GGG ACA CT 3'	587	323 bp
GPX1	Y00433	Forward	5' CCT GGT GGT GCT CGG CTT CC 3'	522	
		Reverse	5' CAA TGG TCT GGA AGC GGC GG 3'	852	350 bp
		CT	5' CAA TGG TCT GGA AGC GGC GGA CCG GAG ACC AGG TGA TGA G 3'	757	279 bp
GPX3	D16360	Forward	5' GCA GAG CCG GGG ACA AGA GAA 3'	113	
		Reverse	5' CTG CTC TTT CTC TCC ATT GAC 3'	471	379 bp
		CT	5' CTG CTC TTT CTC TCC ATT GAC GCT CTT CCT GTA GTG CAT TCA 3'	298	227 bp
GSTM1,2,4,5	J03817	Forward	5' GGG ACG CTC CTG ATT ATG AC 3'	122	
		Reverse	5' GCA AAC CAT GGC CGC TTC CC 3'	442	340 bp
		CT	5' GCA AAC CAT GGC CGC TTC CCT TCT CCA AAA TGT CCA CAC G 3'	301	219 bp
GSTM3	J05459	Forward	5' GTG CGA GTC GTC TAT GGT TC 3'	23	
		Reverse	5' AGT TGT GTG CGG AAA TCC AT 3'	342	339 bp
		CT	5' AGT TGT GTG CGG AAA TCC ATT GCT CTG GGT GAT CTT GTT C 3'	230	247 bp
GSTP1	X08058	Forward	5' TCC GCT GCA AAT ACA TCT CC 3'	305	
		Reverse	5' TGT TTC CCG TTG CCA TTG AT 3'	616	331 bp
		CT	5' TGT TTC CCG TTG CCA TTG ATT AGG ACC TCA TGG ATC AGC A 3'	485	220 bp
GSTT1	X79389	Forward	5' GCT CTA CCT GGA CCT GCT GT 3'	12	
		Reverse	5' GGA ACA CAG GGA ACA TCA CC 3'	351	359 bp
		CT	5' GGA ACA CAG GGA ACA TCA CCT AGA GCA GGA TGG CCA CAC T 3'	199	227 bp
GSTZ1	U86529	Forward	5' TCA CCC CCT ACC CTA CCA TCA GC 3'	806	
		Reverse	5' ATT TCA GCG CGG GCA TTC TTT 3'	1267	482 bp
		CT	5' ATT TCA GCG CGG GCA TTC TTT CCG CAT TCT CAT CTC AGC CTC AC 3'	1161	399 bp
mGST1	J03746	Forward	5' GTC GGA GCA CGG ATC TAC CAC A 3'	404	
		Reverse	5' TTC CTC TGC TCC CCT CCT ACC TA 3'	623	242 bp
		CT	5' TTC CTC TGC TCC CCT CCT ACC TAT TTT CAG CAA CCT GTA AGC C 3'	505	144 bp
SOD1	X02317	Forward	5' TGA AGG TGT GGG GAA GCA TTA 3'	153	
		Reverse	5' TTA CAC CAC AAG CCA AAC GAC 3'	492	360 bp
		CT	5' TTA CAC CAC AAG CCA AAC GAC TGA TGC AAT GGT CTC CTG AGA 3'	384	273 bp
XPA	D14533	Forward	5' CTC GGC GAC GGC GGC TGC GGC TAC TGG AG 3'	178	
		Reverse	5' TGT CGG ACT TCC TTT GCT TCT TCT AAT GC 3'	629	480 bp
		CT	5' TGT CGG ACT TCC TTT GCT TCT TCT AAT GCT CTT TTT TCT AAA TCA CAG TCT 3'	487	360 bp
XRCC1	M36089	Forward	5' CCC CTG AAG AGA CCA AAG CA 3'	1906	
		Reverse	5' CCA TTG AAG GCT GTG ACG TA 3'	2241	355 bp
		CT	5' CCA TTG AAG GCT GTG ACG TAT CAG GGA CTG GCA GAT G 3'	2142	276 bp

**Table 3:** Virtually-multiplexed transcript abundance data.

**SUBJECT #**	**GROUP**	**CEBPB**	**CEBPG**	**E2F1**	**E2F3**	**E2F6**	**EVI1**	**CAT**	**ERCC1**	**ERCC2**	**ERCC4**	**ERCC5**
63	NBCI	7.2E+03	6.4E+02	2.7E+02	1.9E+02	3.4E+02	6.1E+01	2.0E+04	1.1E+05	3.8E+03	1.1E+02	4.3E+04
64	NBCI	7.9E+03	1.7E+03	2.0E+03	2.0E+01	1.7E+02	2.5E+01	2.5E+04	3.0E+05	3.4E+03	8.1E+01	2.0E+05
136	NBCI	6.2E+03	2.1E+02	7.0E+02	1.0E-02	5.0E+01	6.0E+01	2.9E+03	7.4E+03	5.9E+02	5.6E+01	1.9E+04
139	NBCI	4.5E+03	3.4E+03	2.3E+04	5.6E+02	1.0E+03	1.0E+03	6.1E+05	1.2E+06	2.2E+04	3.9E+03	4.6E+05
150	NBCI	8.5E+03	7.4E+02	1.6E+02	1.1E+02	4.1E+01	3.2E+02	3.5E+04	1.7E+05	5.7E+03	6.9E+02	7.2E+04
156	NBCI	2.1E+04	1.2E+03	7.5E+02	ND^1^	ND	1.4E+02	1.5E+04	1.8E+05	2.0E+03	2.9E+02	3.0E+04
157	NBCI	2.3E+04	4.1E+03	3.1E+03	2.1E+02	6.1E+02	1.4E+02	3.5E+05	5.6E+05	8.4E+03	1.6E+03	2.1E+05
194	NBCI	6.5E+03	2.1E+03	2.9E+02	2.6E+02	8.5E+02	4.7E+02	4.5E+04	6.1E+05	4.7E+03	4.4E+02	8.9E+04
210	NBCI	1.0E+04	2.1E+03	7.6E+02	4.0E+02	6.1E+02	3.6E+02	7.6E+04	7.6E+04	3.4E+03	7.7E+02	7.2E+04
257	NBCI	1.1E+04	1.8E+03	2.7E+02	8.9E+02	1.7E+03	9.7E+02	1.0E+05	2.6E+05	1.6E+03	7.6E+02	7.4E+04
261	NBCI	7.6E+03	1.3E+03	2.5E+02	1.7E+02	1.6E+02	9.4E+01	4.4E+04	2.7E+05	5.1E+03	4.6E+02	1.3E+05
282	NBCI	6.4E+03	1.2E+03	5.4E+02	4.1E+01	2.9E+01	1.2E+02	4.1E+04	1.0E+05	6.0E+03	8.1E+02	4.0E+04
285	NBCI	2.6E+03	4.4E+02	2.5E+03	1.1E+03	ND	1.2E+02	3.7E+04	9.1E+04	1.6E+03	7.7E+03	1.6E+04
296	NBCI	1.9E+04	1.0E+03	1.0E+03	ND	2.8E+01	ND	6.9E+04	2.5E+05	3.6E+03	1.7E+02	7.1E+04
305	NBCI	1.8E+03	9.1E+01	6.1E+02	ND	8.7E+01	2.6E+02	2.0E+04	4.9E+04	4.3E+02	2.8E+02	1.3E+04
315	NBCI	3.5E+03	1.3E+03	1.2E+03	2.0E+02	7.5E+01	6.1E+02	4.7E+04	2.1E+05	2.7E+03	2.4E+03	6.2E+04
330	NBCI	2.7E+03	2.4E+02	4.0E+02	1.1E+02	3.5E+02	4.0E+02	3.6E+04	8.3E+04	1.5E+03	1.7E+03	2.8E+04
331	NBCI	7.3E+03	1.3E+03	8.0E+02	ND	ND	ND	8.6E+04	2.6E+05	6.5E+02	6.4E+02	6.2E+04
334	NBCI	3.7E+03	6.1E+02	1.1E+03	2.3E+01	1.9E+01	4.0E+01	7.8E+04	1.4E+05	2.5E+03	1.3E+03	5.1E+04
336	NBCI	3.6E+03	8.4E+02	2.8E+03	4.3E+02	1.2E+02	2.5E+02	8.9E+04	2.1E+05	7.9E+03	3.1E+03	9.5E+04
337	NBCI	5.0E+03	9.3E+02	1.1E+03	1.6E+02	2.5E+02	ND	6.5E+04	1.9E+05	3.3E+03	1.3E+03	4.2E+04
339	NBCI	2.5E+03	3.2E+02	5.2E+02	3.4E+01	4.3E+01	6.3E+01	5.0E+04	8.7E+04	2.3E+03	7.7E+02	3.2E+04
361	NBCI	7.9E+03	2.5E+03	6.7E+02	5.8E+02	1.4E+03	3.5E+02	6.7E+04	2.5E+05	1.5E+03	2.8E+03	4.7E+04
363	NBCI	6.2E+03	1.7E+03	1.4E+03	1.1E+02	1.9E+02	5.2E+01	7.6E+04	1.4E+05	3.7E+03	7.3E+02	7.2E+04

34	BCI	1.9E+03	1.7E+03	5.7E+02	2.4E+02	1.2E+02	3.7E+02	6.7E+04	5.6E+05	6.1E+03	1.5E+03	1.2E+05
71	BCI	7.6E+03	1.2E+03	1.3E+03	1.7E+01	1.8E+02	2.1E+01	9.5E+04	6.7E+05	1.8E+04	2.4E+02	2.2E+05
85	BCI	1.0E+04	9.7E+02	8.7E+02	6.3E+01	ND	ND	2.6E+04	4.9E+04	1.1E+03	3.9E+02	2.3E+04
88	BCI	1.5E+03	4.0E+02	4.2E+02	1.1E+02	ND	ND	3.1E+04	2.9E+04	2.7E+03	3.6E+02	3.9E+04
99	BCI	1.4E+04	3.3E+03	2.6E+03	2.2E+02	3.1E+03	1.4E+02	2.1E+05	2.0E+05	3.7E+03	9.3E+02	1.4E+05
118	BCI	1.5E+03	1.2E+03	2.8E+02	ND	ND	1.4E+02	2.4E+04	3.5E+04	1.9E+03	1.2E+03	1.7E+04
146	BCI	1.9E+04	1.2E+03	2.3E+02	1.2E+02	1.6E+03	6.0E+01	3.1E+04	1.4E+05	4.9E+03	2.7E+02	6.2E+04
147	BCI	2.8E+03	1.3E+03	N/A^2^	3.6E+02	2.8E+02	3.8E+02	6.1E+04	3.0E+05	2.7E+03	1.1E+03	1.8E+04
158	BCI	4.9E+03	8.8E+02	1.9E+02	2.8E+01	4.0E+02	6.8E+01	2.2E+04	1.4E+05	1.7E+03	1.5E+02	1.3E+04
167	BCI	6.7E+03	9.2E+02	4.2E+02	1.8E+02	8.2E+02	3.8E+02	8.9E+04	1.1E+05	2.2E+03	2.8E+02	4.1E+04
171	BCI	1.0E+04	3.9E+03	3.2E+03	5.1E+02	8.9E+01	2.4E+02	5.3E+05	8.3E+05	1.3E+04	6.3E+02	1.6E+05
191	BCI	1.6E+04	2.4E+03	1.5E+02	1.5E+02	7.1E+01	1.2E+02	2.0E+04	9.6E+04	2.5E+03	3.4E+02	1.2E+04
211	BCI	3.8E+03	1.1E+03	6.5E+02	6.3E+02	3.2E+02	2.9E+02	8.3E+04	3.5E+05	2.8E+03	3.2E+02	9.1E+04
212	BCI	1.8E+04	2.8E+03	6.0E+02	3.4E+02	2.0E+02	2.2E+02	3.4E+04	2.1E+05	5.2E+03	3.0E+02	3.6E+04
247	BCI	6.5E+03	7.5E+02	6.4E+02	1.8E+02	ND	1.2E+02	7.8E+04	4.4E+04	5.5E+02	2.7E+03	1.3E+04
255	BCI	1.9E+04	1.1E+03	6.1E+02	1.8E+01	2.3E+02	7.6E+01	1.1E+05	6.5E+05	1.2E+04	1.2E+03	3.8E+05
259	BCI	8.4E+03	6.4E+02	5.1E+02	ND	6.7E+01	4.0E+01	4.7E+04	1.5E+05	2.6E+03	3.8E+02	7.9E+04
271	BCI	4.1E+03	6.6E+02	1.4E+03	6.5E+01	2.7E+02	1.9E+02	9.1E+04	1.5E+05	1.6E+03	ND	1.5E+05
287	BCI	8.7E+03	1.1E+03	9.5E+01	7.4E+01	4.0E+01	2.6E+02	4.7E+04	1.2E+05	6.5E+03	1.1E+02	4.1E+04
300	BCI	4.9E+03	4.4E+02	4.1E+02	4.4E+01	ND	5.1E+01	3.4E+04	6.9E+04	1.0E+03	1.0E+03	2.6E+04
306	BCI	6.5E+03	6.8E+02	4.9E+02	4.4E+01	ND	1.7E+01	3.7E+04	2.5E+05	2.8E+03	3.2E+02	4.1E+04
314	BCI	4.4E+03	9.3E+02	4.8E+02	3.1E+02	ND	1.9E+02	5.3E+04	6.2E+04	4.5E+03	5.2E+02	3.1E+04
329	BCI	1.4E+04	3.5E+02	2.4E+02	ND	ND	ND	7.9E+04	1.5E+05	1.4E+03	ND	1.5E+05
335	BCI	4.2E+03	3.7E+02	2.2E+03	3.3E+02	2.4E+02	2.4E+02	4.8E+04	1.9E+05	9.8E+03	3.1E+02	3.3E+04
B3	BCI	7.4E+03	9.3E+02	1.4E+02	2.4E+02	1.8E+02	3.8E+02	3.4E+04	1.6E+05	2.2E+03	2.8E+02	4.1E+04

**SUBJECT #**	**GROUP**	**GPX1**	**GPX3**	**GSTM15**	**GSTM3**	**GSTP1**	**GSTT1**	**GSTZ1**	**MGST1**	**SOD1**	**XPA**	**XRCC1**

63	NBCI	8.4E+05	1.5E+03	7.3E+03	3.9E+03	1.9E+06	ND	3.1E+03	1.7E+05	1.6E+05	2.5E+03	2.1E+04
64	NBCI	4.4E+05	3.1E+02	1.5E+04	2.6E+03	3.5E+06	6.4E+03	2.0E+03	1.6E+05	6.5E+05	2.2E+03	4.2E+04
136	NBCI	2.0E+05	5.2E+02	6.3E+03	2.2E+03	5.3E+05	ND	8.9E+02	3.2E+04	5.3E+04	1.1E+03	4.4E+03
139	NBCI	1.8E+06	3.2E+03	5.2E+04	8.3E+03	3.2E+07	ND	2.8E+04	8.4E+05	2.2E+06	2.6E+04	1.6E+05
150	NBCI	2.1E+05	2.8E+03	1.1E+04	1.1E+03	1.5E+06	ND	3.1E+03	3.1E+04	2.0E+05	2.0E+03	3.1E+04
156	NBCI	4.8E+05	3.9E+03	1.9E+04	6.5E+03	2.9E+06	ND	4.9E+03	5.8E+04	1.3E+05	4.0E+03	2.5E+04
157	NBCI	2.4E+06	2.0E+03	2.6E+04	3.3E+03	1.8E+07	8.5E+03	3.6E+03	3.6E+05	1.8E+06	6.2E+03	1.7E+05
194	NBCI	5.4E+05	4.0E+03	1.2E+04	1.1E+03	7.8E+06	7.7E+03	3.2E+03	9.6E+04	5.8E+05	3.7E+03	2.4E+05
210	NBCI	3.2E+05	3.3E+03	4.1E+03	2.6E+03	3.9E+06	1.8E+03	3.7E+03	7.2E+04	3.3E+05	4.8E+03	2.9E+04
257	NBCI	6.3E+05	2.5E+03	1.1E+04	1.2E+02	2.8E+06	8.7E+03	2.9E+03	2.3E+04	1.2E+05	2.6E+03	4.9E+04
261	NBCI	7.6E+05	4.7E+03	1.6E+04	7.4E+03	3.1E+06	1.5E+03	1.8E+03	1.2E+05	4.5E+05	4.9E+03	7.2E+04
282	NBCI	4.6E+05	2.5E+03	1.3E+04	2.3E+03	1.9E+06	1.5E+04	1.3E+03	6.5E+04	4.5E+05	2.0E+03	3.1E+04
285	NBCI	4.3E+05	5.3E+03	8.4E+04	6.2E+02	3.5E+06	7.3E+03	9.2E+03	3.0E+04	2.3E+05	ND	2.0E+04
296	NBCI	8.1E+05	1.5E+03	7.4E+03	4.1E+03	3.6E+06	8.1E+03	4.5E+03	2.0E+05	5.4E+05	2.6E+03	5.2E+04
305	NBCI	1.4E+05	6.5E+02	4.0E+03	3.2E+03	1.6E+06	ND	6.9E+02	4.1E+04	1.8E+05	1.3E+03	5.7E+03
315	NBCI	4.2E+05	7.6E+03	5.1E+03	3.1E+03	7.9E+06	7.2E+03	4.3E+03	7.3E+04	4.5E+05	1.0E+04	3.8E+04
330	NBCI	1.2E+05	2.0E+03	5.8E+03	3.5E+03	1.1E+06	3.1E+01	1.3E+03	2.9E+04	2.3E+05	1.4E+03	1.7E+04
331	NBCI	6.1E+05	3.4E+03	1.3E+04	3.6E+03	7.3E+06	1.0E+04	1.8E+03	1.2E+05	1.3E+06	1.9E+03	5.7E+04
334	NBCI	6.5E+05	4.0E+03	2.7E+04	1.7E+04	3.7E+06	4.6E+03	3.0E+03	6.8E+04	5.9E+05	2.9E+03	2.6E+04
336	NBCI	4.7E+05	2.7E+03	4.4E+04	1.6E+03	3.4E+06	1.2E+04	8.3E+03	6.2E+04	5.4E+05	4.2E+03	1.2E+05
337	NBCI	2.8E+05	2.8E+03	3.8E+03	4.3E+03	1.5E+06	5.3E+03	5.6E+03	4.9E+04	3.3E+05	1.7E+03	4.4E+04
339	NBCI	3.1E+05	1.6E+04	3.0E+04	1.1E+03	3.5E+06	6.6E+03	2.0E+03	6.6E+04	2.4E+05	2.5E+03	2.2E+04
361	NBCI	3.7E+05	8.4E+02	1.5E+04	1.3E+03	7.8E+06	3.1E+03	2.7E+03	4.8E+04	6.4E+05	6.1E+03	7.7E+04
363	NBCI	6.1E+05	2.7E+03	2.2E+04	2.0E+03	8.1E+06	9.9E+03	2.4E+03	9.7E+04	7.0E+05	7.0E+03	3.6E+04

34	BCI	8.8E+05	3.1E+03	3.4E+03	5.1E+02	1.8E+06	ND	1.9E+03	6.1E+04	2.9E+05	3.1E+03	5.6E+04
71	BCI	8.8E+05	1.3E+04	3.2E+04	2.2E+03	6.7E+06	ND	2.5E+03	4.1E+05	7.5E+05	3.1E+03	5.6E+05
85	BCI	2.3E+05	1.1E+03	2.6E+04	4.1E+03	1.2E+06	1.0E+04	8.1E+02	4.8E+04	1.5E+05	3.5E+03	1.8E+04
88	BCI	1.3E+05	1.5E+03	3.5E+03	1.1E+03	6.9E+05	ND	5.6E+02	2.4E+04	1.9E+05	2.4E+03	1.6E+04
99	BCI	9.0E+05	2.1E+03	1.5E+04	5.5E+03	9.6E+06	6.7E+03	8.0E+03	1.2E+05	9.4E+05	8.5E+03	4.7E+04
118	BCI	8.0E+04	3.8E+03	1.1E+04	4.8E+03	1.7E+06	4.0E+03	4.5E+02	4.4E+04	1.7E+05	1.1E+03	6.1E+03
146	BCI	4.1E+05	2.9E+04	4.7E+04	4.7E+02	2.0E+06	7.1E+03	1.6E+04	6.6E+04	1.7E+05	3.8E+04	6.5E+04
147	BCI	4.1E+05	2.5E+03	8.2E+03	7.3E+02	8.8E+05	1.7E+03	1.3E+03	2.3E+04	6.5E+05	2.3E+02	7.4E+04
158	BCI	2.6E+04	2.6E+03	1.4E+04	4.7E+03	2.3E+06	2.9E+03	1.2E+03	2.3E+04	3.7E+04	1.9E+03	2.3E+04
167	BCI	1.9E+05	2.5E+03	5.3E+03	1.8E+03	2.4E+06	1.4E+04	2.3E+03	9.5E+04	4.0E+05	2.9E+03	2.8E+04
171	BCI	1.6E+06	1.7E+03	1.6E+04	4.0E+03	7.8E+06	6.0E+03	6.5E+03	2.5E+05	1.4E+06	1.4E+04	6.0E+04
191	BCI	4.5E+05	1.9E+03	3.5E+03	2.5E+02	1.7E+06	3.6E+03	5.6E+02	2.1E+04	1.6E+05	2.5E+03	2.1E+04
211	BCI	6.0E+05	1.7E+04	1.1E+04	1.3E+02	1.0E+07	5.5E+03	4.1E+03	2.4E+04	7.1E+05	4.3E+03	8.3E+04
212	BCI	6.5E+04	1.6E+03	9.6E+03	1.1E+03	4.0E+06	8.1E+03	9.5E+02	7.5E+04	9.4E+04	3.1E+03	3.6E+04
247	BCI	2.3E+05	2.0E+02	1.4E+04	2.1E+03	1.3E+06	7.1E+03	1.1E+03	2.4E+04	1.3E+05	1.3E+03	1.1E+04
255	BCI	1.3E+06	7.3E+03	1.5E+04	2.5E+03	2.8E+06	3.8E+04	8.1E+03	1.1E+05	2.8E+06	2.0E+03	1.0E+05
259	BCI	3.0E+06	5.9E+03	1.4E+04	7.0E+03	9.2E+06	5.1E+03	5.1E+03	1.1E+05	5.0E+05	4.8E+03	2.1E+04
271	BCI	7.4E+05	9.5E+02	3.8E+03	2.6E+03	2.4E+06	3.7E+03	3.1E+03	7.1E+04	6.3E+05	4.1E+03	3.0E+04
287	BCI	5.8E+05	8.7E+03	1.1E+04	2.3E+03	1.9E+06	1.5E+04	6.3E+02	8.8E+04	1.2E+05	4.7E+03	3.3E+04
300	BCI	2.7E+05	1.5E+03	7.7E+03	5.0E+03	3.7E+06	7.1E+03	1.9E+03	6.7E+04	3.2E+05	8.0E+02	1.3E+04
306	BCI	4.2E+05	2.0E+02	9.7E+03	2.3E+03	2.2E+06	ND	4.8E+03	5.6E+04	1.1E+06	4.5E+02	1.9E+04
314	BCI	1.6E+05	3.7E+03	1.7E+04	1.1E+03	1.2E+06	1.9E+02	1.5E+03	2.7E+04	2.7E+05	2.8E+03	3.4E+04
329	BCI	2.3E+05	8.9E+03	1.6E+04	5.3E+03	7.2E+06	7.8E+03	1.4E+03	5.7E+04	8.9E+05	1.9E+03	4.8E+04
335	BCI	4.1E+05	6.5E+03	2.1E+04	7.1E+03	4.3E+06	1.8E+02	7.0E+03	6.9E+04	5.2E+05	3.9E+03	4.4E+04
B3	BCI	4.0E+05	3.2E+03	6.0E+03	3.8E+02	1.6E+06	7.7E+03	1.8E+03	1.8E+04	2.6E+05	3.7E+03	1.7E+04

Virtually-multiplexed transcript abundance data for each gene (in the form of molecules/10^6 ^β-actin molecules) from all experiments were included in the same Standardized Expression Database (SED). These data are now directly comparable to previously published virtually-multiplexed transcript abundance data from this laboratory [15], or to Virtually-multiplexed transcript abundance data collected by others who use the NCI-funded (R24 CA 95806) Standardized Expression Measurement (SEM) Center. The data presented here represent more than 6,000 Virtually-multiplexed transcript abundance measurements conducted in multiple experiments. The sixteen antioxidant or DNA repair genes and each of the six transcription factors except for E2F1 were measured in each NBEC sample from 49 individuals (24 non-BC individuals and 25 BC individuals).

^1^ND, Not detectable (When 60 molecules of Competitive Template (CT) were added to the reaction, CT was detected but native template was not.)

^2^NA, Not available. E2F1 was measured in all of the samples except for one BC individual (24 non-BC individuals and 24 BC individuals).

**Table 4:** Bivariate analysis of virtually-multiplexed transcript abundance data values for each antioxidant or DNA repair gene versus each transcription factor.

	Non-BC Individuals n = 24		BC Individuals n = 25		ALL n = 49	
Antioxidant/DNA Repair Genes vs Transcription Factors	*r *Value	p Value	*r *Value	p Value	*r *Value	p Value

CAT vs CEBPB	0.13	1	0.18	1	0.15	1
CAT vs CEBPG	0.65	**0.004**	0.35	0.48	0.55	**<0.0006**
CAT vs E2F1*	0.54	0.04	0.68	**0.002**	0.56	**<0.0006**
CAT vs E2F3	0.48	0.12	0.18	1	0.37	0.06
CAT vs E2F6	0.26	1	0.3	0.84	0.25	0.48
CAT vs EVI1	-0.01	1	0.21	1	0.08	1
ERCC1 vs CEBPB	0.32	0.78	0.27	1	0.29	0.24
ERCC1 vs CEBPG	0.77	**<0.0006**	0.42	0.24	0.62	**<0.0006**
ERCC1 vs E2F1	0.35	0.54	0.39	0.36	0.37	0.06
ERCC1 vs E2F3	0.39	0.36	0.21	1	0.31	0.18
ERCC1 vs E2F6	0.17	1	0.63	**0.005**	0.42	0.02
ERCC1 vs EVI1	-0.02	1	0.38	0.36	0.17	1
ERCC2 vs CEBPB	0.25	1	0.19	1	0.22	0.84
ERCC2 vs CEBPG	0.63	**0.006**	0.39	0.3	0.53	**<0.0006**
ERCC2 vs E2F1	0.39	0.36	0.32	0.72	0.33	0.12
ERCC2 vs E2F3	0.58	0.02	0.22	1	0.42	0.02
ERCC2 vs E2F6	0.37	0.42	0.51	0.06	0.42	0.02
ERCC2 vs EVI1	0.19	1	0.29	0.96	0.23	0.66
ERCC4 vs CEBPB	-0.35	0.6	-0.11	1	-0.16	1
ERCC4 vs CEBPG	0.24	1	0.37	0.42	0.25	0.48
ERCC4 vs E2F1	0.42	0.24	0.04	1	0.2	1
ERCC4 vs E2F3	0.6	**0.01**	0.33	0.6	0.33	0.12
ERCC4 vs E2F6	-0.04	1	0.04	1	0.07	1
ERCC4 vs EVI1	0.24	1	0.33	0.66	0.27	0.36
ERCC5 vs CEBPB	0.4	0.3	0.28	1	0.33	0.12
ERCC5 vs CEBPG	0.79	**<0.0006**	0.12	1	0.46	**0.005**
ERCC5 vs E2F1	0.44	0.18	0.45	0.18	0.44	**0.01**
ERCC5 vs E2F3	0.39	0.36	-0.11	1	0.13	1
ERCC5 vs E2F6	0.41	0.3	0.35	0.54	0.38	0.04
ERCC5 vs EVI1	0.07	1	-0.04	1	0.01	1
GPX1 vs CEBPB	0.49	0.06	0.24	1	0.32	0.12
GPX1 vs CEBPG	0.72	**<0.0006**	0.19	1	0.4	0.02
GPX1 vs E2F1	0.48	0.12	0.38	0.36	0.43	0.02
GPX1 vs E2F3	0.22	1	-0.004	1	0.08	1
GPX1 vs E2F6	0.06	1	0.36	0.48	0.28	0.3
GPX1 vs EVI1	-0.06	1	0.2	1	0.1	1
GPX3 vs CEBPB	-0.18	1	0.14	1	0.02	1
GPX3 vs CEBPG	0.13	1	0.01	1	0.07	1
GPX3 vs E2F1	-0.03	1	-0.17	1	-0.12	1
GPX3 vs E2F3	0.32	0.78	-0.2	1	0.05	1
GPX3 vs E2F6	-0.26	1	0.44	0.18	0.19	1
GPX3 vs EVI1	0.06	1	0.08	1	0.06	1
GSTM1-5 vs CEBPB	-0.08	1	0.43	0.18	0.17	1
GSTM1-5 vs CEBPG	0.25	1	0.02	1	0.16	1
GSTM1-5 vs E2F1	0.51	0.06	0.23	1	0.41	0.02
GSTM1-5 vs E2F3	0.29	0.96	-0.16	1	0.1	1
GSTM1-5 vs E2F6	-0.3	0.9	0.006	1	-0.1	1
GSTM1-5 vs EVI1	0.22	1	-0.12	1	0.07	1
GSTM3 vs CEBPB	0.01	1	0.06	1	0.04	1
GSTM3 vs CEBPG	-0.007	1	-0.28	1	0.01	1
GSTM3 vs E2F1	0.29	1	0.35	0.54	0.34	0.12
GSTM3 vs E2F3	-0.31	0.84	-0.49	0.06	-0.4	0.03
GSTM3 vs E2F6	-0.11	1	-0.25	1	-0.16	1
GSTM3 vs EVI1	-0.27	1	-0.25	1	-0.25	0.54
GSTP1 vs CEBPB	0.19	1	0.38	0.36	0.28	0.36
GSTP1 vs CEBPG	0.74	**<0.0006**	0.18	1	0.51	**0.001**
GSTP1 vs E2F1	0.6	**0.01**	0.46	0.12	0.56	**<0.0006**
GSTP1 vs E2F3	0.32	0.78	-0.25	1	0.07	1
GSTP1 vs E2F6	0.1	1	0.35	0.48	0.26	0.42
GSTP1 vs EVI1	0.11	1	0.13	1	0.12	1
GSTT1 vs CEBPB	0.03	1	0.45	0.12	0.24	0.6
GSTT1 vs CEBPG	0.39	0.36	0.16	1	0.3	0.24
GSTT1 vs E2F1	0.07	1	-0.15	1	-0.05	1
GSTT1 vs E2F3	0.35	0.54	-0.1	1	0.17	1
GSTT1 vs E2F6	0.05	1	0.21	1	0.11	1
GSTT1 vs EVI1	-0.26	1	0.22	1	-0.04	1
GSTZ1 vs CEBPB	0.11	1	0.36	0.42	0.25	0.54
GSTZ1 vs CEBPG	0.51	0.06	0.08	1	0.28	0.3
GSTZ1 vs E2F1	0.64	**0.004**	0.5	0.06	0.58	**<0.0006**
GSTZ1 vs E2F3	0.42	0.24	0.14	1	0.25	0.54
GSTZ1 vs E2F6	-0.05	1	0.48	0.12	0.32	0.18
GSTZ1 vs EVI1	0.02	1	0.27	1	0.16	1
mGST vs CEBPB	0.31	0.78	0.35	0.48	0.32	0.12
mGST vs CEBPG	0.56	0.02	0.25	1	0.42	0.02
mGST vs E2F1	0.58	0.02	0.54	0.04	0.58	**<0.0006**
mGST vs E2F3	0.03	1	-0.15	1	-0.06	1
mGST vs E2F6	0.17	1	0.29	0.96	0.27	0.36
mGST vs EVI1	-0.16	1	0.07	1	-0.04	1
SOD1 vs CEBPB	0.13	1	0.15	1	0.14	1
SOD1 vs CEBPG	0.66	**0.002**	0.009	1	0.36	0.06
SOD1 vs E2F1	0.59	0.02	0.55	0.04	0.56	**<0.0006**
SOD1 vs E2F3	0.25	1	-0.07	1	0.09	1
SOD1 vs E2F6	0.12	1	0.14	1	0.14	1
SOD1 vs EVI1	-0.17	1	0.03	1	-0.06	1
XPA vs CEBPB	0.31	0.84	0.42	0.24	0.31	0.18
XPA vs CEBPG	0.36	0.54	0.33	0.66	0.34	0.12
XPA vs E2F1	-0.05	1	0.22	1	-0.02	1
XPA vs E2F3	-0.07	1	0.14	1	-0.01	1
XPA vs E2F6	0.55	0.04	0.46	0.12	0.4	0.02
XPA vs EVI1	0.04	1	0.07	1	0.04	1
XRCC1 vs CEBPB	0.36	0.48	0.28	1	0.32	0.18
XRCC1 vs CEBPG	0.83	**<0.0006**	0.27	1	0.591	**<0.0006**
XRCC1 vs E2F1	0.32	0.78	0.37	0.48	0.35	0.12
XRCC1 vs E2F3	0.47	0.12	0.22	1	0.35	0.06
XRCC1 vs E2F6	0.26	1	0.54	0.04	0.41	0.02
XRCC1 vs EVI1	-0.009	1	0.12	1	0.06	^1^

The correlation of logarithmically transformed virtually-multiplexed transcript abundance data (Table 3) from each of the six transcription factors with each of the sixteen antioxidant or DNA repair genes was determined. The transformation was necessary due to the wide range of expression of each gene among the samples. The correlation coefficient (*r *value) and level of significance (p value) for each correlation are presented. Significance (p < 0.01) was determined using a two-tailed test following Bonferroni adjustment for six multiple comparisons (comparison of each of six transcription factors to each of the antioxidant or DNA repair genes).

*values for E2F1 obtained in all but one BC individuals.
